# A study of specific immunoglobulin G4 expression in allergic rhinitis and its value in assessing efficacy and in predicting prognosis of sublingual immunotherapy

**DOI:** 10.1002/kjm2.12916

**Published:** 2024-12-30

**Authors:** Ting‐Ting Wei, Kai Gao, Jun‐Hu Tai, Yong‐Jun Wei, Bin Zhan

**Affiliations:** ^1^ Department of Otorhinolaryngology Head and Neck Surgery The Second Affiliated Hospital of Xiamen Medical College Xiamen Fujian Province China

**Keywords:** allergic rhinitis, specific immunoglobulin G4, sublingual immunotherapy, value of prognostic prediction, value of treatment efficacy assessment

## Abstract

Allergic rhinitis (AR) is a widespread health issue with a rising global prevalence, and sublingual immunotherapy (SLIT) has shown efficacy in AR treatment. We examined specific immunoglobulin G4 (sIgG4) expression in AR and its role in evaluating SLIT efficacy and predicting patient prognosis. We compared total nasal symptom score (TNSS), total medication score (TMS), visual analogue scale (VAS) score, inflammatory cytokines, and immune function markers in AR patients before and after SLIT. SLIT reduced TNSS, TMS, VAS scores, IL‐4, IL‐17, eosinophilia percentage (EOS%), and specific immunoglobulin E (sIgE) levels, while increasing INF‐γ, IL‐10, and sIgG4. The sIgG4 level at pre‐treatment and 12 months post‐treatment was negatively correlated with TNSS, TMS, VAS score, IL‐4, IL‐17, EOS%, and sIgE, and positively correlated with IFN‐γ and IL‐10. Most patients showed symptomatic improvement. After 12 months, sIgG4 level demonstrated an area under the curve (AUC) of 0.867 for assessing SLIT as effective. Pre‐treatment sIgG4 level showed an AUC of 0.869 for predicting SLIT as effective. Collectively, sIgG4 has strong potential assessing SLIT efficacy and prognosis in AR patients, with correlations to TNSS, TMS, VAS score, and IL‐4, IL‐10, IL‐17, INF‐γ, EOS% and sIgE levels.

## 1 INTRODUCTION

Allergic rhinitis (AR) is a chronic condition that affects roughly 40% of the global population, presenting with symptoms such as sneezing, postnasal drip, rhinorrhea, and nasal congestion.^1^, ^2^, ^3^ Due to its impact on the upper and lower respiratory tracts, AR often leads to complications, including a significant contribution to uncontrolled asthma.^4^ Currently, specific immunotherapy targets the underlying mechanisms of immunoglobulin E (IgE)‐mediated type I allergic responses, promoting immune tolerance through allergen extracts and reducing symptoms upon allergen reexposure.^5^ In China, allergists generally consider sublingual immunotherapy (SLIT) an effective treatment option for AR and asthma, given its strong safety profile and tolerability.^6^

The effectiveness of SLIT in AR is commonly assessed through subjective measures, including the total nasal symptom score (TNSS), total medication score (TMS), and visual analogue scale (VAS) score.^7^, ^8^ However, these tools depend heavily on patients' subjective perceptions, which can be influenced by various factors, such as age, sex, education, and socioeconomic status, leading to potential bias.^9^ Consequently, there is an urgent need for objective immunologic indicators to assess and predict treatment responses in AR patients, providing a more accurate basis for treatment guidance.

AR is triggered by disruptions in the epithelial barrier, allowing allergens to penetrate the nasal mucosa and activate a T‐helper type 2 inflammatory response, resulting in allergen‐specific IgE (sIgE) production.^10^ Specific immunoglobulin G4 (sIgG4) has been shown to act as a blocking antibody to IgE, binding competitively to mast cell receptors and thereby mitigating allergic response.^11^ Elevated sIgG4 levels have been associated with symptom relief in AR, and sIgG4 has emerged as a potential immunological marker for assessing treatment efficacy.^12^, ^13^ For example, subcutaneous immunotherapy with house dust mite allergens has been linked to increased sIgG4 levels and a corresponding decrease in Th2 cytokines in house dust mite‐allergic asthma patients.^14^ It is also hypothesized that sustained elevation of sIgG4 contributes to symptom improvement in SLIT‐treated AR patients.^15^

However, the relationship between sIgG4 expression and clinical efficacy, as well as its potential as an objective marker for evaluating and predicting SLIT outcomes, remains debated. This study aimed to analyze sIgG4 expression in AR and assess its value in evaluating SLIT efficacy and predicting patient prognosis.

## 2 MATERIALS AND METHODS

### 2.1 Ethics statement

The study was reviewed and approved by the Academic Ethics Committee of The Second Affiliated Hospital of Xiamen Medical College and was conducted in compliance with the Declaration of Helsinki.

### 2.2 Study subjects

From January 2021 to May 2023, a total of 148 patients with AR were assessed at The Second Affiliated Hospital of Xiamen Medical College. Based on inclusion and exclusion criteria, 100 patients (55 males and 45 females) were ultimately included in the study. The participants ranged in age from 18 to 41 years, with a mean age of 28.5 years, and their disease duration ranged from 1 to 8 years, with a mean disease course of 4.6 years.

### 2.3 Inclusion and exclusion criteria

The inclusion criteria were as follows: (1) met the diagnostic criteria outlined in the Allergic Rhinitis and its Impact on Asthma guidelines,^16^ with the diagnosis of AR confirmed via symptoms, physical examination, skin prick test (SPT), and sIgE test; (2) presented symptoms such as nasal itching, nasal congestion, paroxysmal sneezing, runny nose; (3) positive response (++ or higher) to 50 mg/mL mite extract in SPT; (4) UniCAP test result of grade 2 or higher for dust mite‐sIgE in serum; (5) no participation in any other clinical trials within a month prior to admission; (6) Age over 18 years; (7) complete clinical data; and (8) received SLIT treatment.

The exclusion criteria were as follows: allergy to allergens other than dust mites, comorbid asthma or nasal comorbid, acute infections or fever, severe dysfunctions in vital organs (heart, liver or kidneys), immune system disorders, mental illness, pregnancy, or lactation.

### 2.4 Clinical administration method

Patients underwent SLIT with dust mite drops (S20060012, Changdi, Wolwopharma, Huzhou, Zhejiang, China) alongside symptomatic treatment. The SLIT protocol included: Week 1: Changdi No.1 (1 μg/mL) administered in progressively increasing dosages of 1, 2, 3, 4, 6, 8, and 10 drops on days 1–7, respectively; Week 2: Changdi No.2 (10 μg/ml) administered in the same dosage pattern as Week 1; Week 3: Changdi No.3 (100 μg/mL), also following the same dosage pattern as Week 1; Weeks 4 and 5: Changdi No. 4 (333 μg/ml) at 3 drops once daily; Week 6 onward: Changdi No.5 (1000 μg/mL) was administered as a maintenance dose until the end of the 12‐month treatment period. Each patient completed a full 12‐month SLIT regimen.

### 2.5 Indicator observation

Relevant clinical data, including age, sex, disease duration, and allergen test results, were obtained from patients' electronic medical records. TNSS, TMS, and VAS scores, along with inflammatory markers and immune‐related indicators, were recorded at 3, 6, 9, and 12 months of treatment.

1. TNSS was calculated based on four symptoms: (a) Nasal congestion: 3 points for mouth breathing almost all day; 2 points for intermittent or alternating mouth breathing; 1 point for mild symptoms; 0 points for no symptoms. (b) Nasal itching: 3 points for intolerable itching; 2 points for tolerable itching; 1 point for intermittent itching; 0 points for no symptoms. (c) Sneezing: 3 points for 11 or more consecutive sneezes, 2 points for 6–10 sneezes, 1 point for 3–5 sneezes, and 0 points for fewer than 3 sneezes. (d) Runny nose: 3 points for 10 or more nose blows per day, 2 points for 5–9 blows, 1 point for 4 or fewer blows, and 0 points for no symptoms. The TNSS was the total of these four nasal symptom scores.
2. TMS was calculated as follows: (a) 3 points for oral glucocorticoids; (b) 2 points for topical glucocorticoids (nasal or inhaled); 1 point each for bronchodilators; antileukotrienes, oral antihistamines, and nasal antihistamines.

The total cumulative score was recorded as the TMS.

3 VAS involved a 10 cm scale marked from “0” (no symptoms) to “10” (extreme symptoms/discomfort). Patients indicated their symptom severity on this scale, with the physician assessing the score based on the indicated position.
4 Serum levels of interferon‐γ (IFN‐γ; EK180), interleukin (IL)‐4 (EK104), IL‐10 (EK110), and IL‐17 (EK1177) were measured using enzyme‐linked immunosorbent assay (ELISA)^17^, ^18^ kits from Multi Sciences (Hangzhou, Zhejiang, China). Eosinophil (EOS) counts were determined using a UniCel DxH 800 analyzer (Beckman Coulter, Chaska, MN, USA).^19^ Dust mite sIgG4 and sIgE levels were assessed using the ImmunoCAP detection system (ThermoFisher Scientific, Phadia, Uppsala, Sweden).^20^

### 2.6 Clinical efficacy

Clinical efficacy was evaluated based on the guidelines from the Expert Consensus on Allergen‐Specific Immunotherapy for Allergic Rhinitis. The efficacy index was calculated as follows:

Efficacy index = (pre‐treatment TNSS score − post‐treatment TNSS score)/pre‐treatment TNSS score × 100% (markedly effective ≥50%; effective 20%–50%; ineffective <20%). The total effective rate was defined as: Total effective rate = (markedly effective cases + effective cases)/total number of cases × 100%.

### 2.7 Statistical analysis

Data were statistically analyzed and graphed using SPSS 27.0 (BMI, Armonk, NY) and GraphPad Prism 9.5 (GraphPad Software, San Diego, CA). The Kolmogorov–Smirnov test used to evaluate the normality of data distribution. For normally distributed data, results were presented as mean ± SD, and repeated measures analysis of variance (ANOVA) was used for comparisons at different time points within groups. Non‐normally distributed data were reported as median (minimum, maximum), with the Kruskal–Wallis test applied for comparisons between groups. Categorical variables were expressed as frequencies and percentages (%), and intergroup comparisons were performed using the χ^2^ test. Spearman correlation analysis assessed the associations of sIgG4 with TNSS, TMS, and VAS scores, as well as with inflammatory and immune function markers. For normally distributed data, Pearson's correlation coefficient was used. The diagnostic value of IL‐4, IL‐17, INF‐γ, IL‐10, EOS, sIgE, and sIgG4 levels for assessing SLIT efficacy after 12 months of treatment, as well as the predictive value of pre‐treatment sIgG4 levels of SLIT efficacy prognosis, was separately evaluated using receiver operating characteristic (ROC) curves. Statistical significance was determined at *p* <0.05 in two‐sided tests.

## 3 RESULTS

### 3.1 Comparisons of pre‐ and post‐treatment TNSS, TMS, and VAS scores in AR patients

TNSS, TMS and VAS scores were measured in AR patients before SLIT treatment and 3, 6, 9, and 12 months post‐treatment. As shown in Table 1, there was a significant reduction in TNSS, TMS and VAS scores at each time point compared to baseline (all *p* <0.001), indicating that SLIT effectively alleviated clinical symptoms in AR patients.

**TABLE 1:** Comparisons of TNSS, TMS, and VAS scores of AR patients before and after treatment.

	TNSS score	TMS score	VAS score
Before treatment	6 (4,10)	3 (1,4)	8 (5,10)
3 months post‐treatment	6 (3,9)	2 (1,3)	7 (3,9)
*P*_1_	<0.001	<0.001	<0.001
6 months post‐treatment	5 (2,9)	2 (1,3)	6 (3,8)
*P*_2_	<0.001	<0.001	<0.001
9 months post‐treatment	4 (1,8)	1.5 (0,3)	5 (2,7)
*P*_3_	<0.001	<0.001	<0.001
12 months post‐treatment	2 (0,5)	1 (0,3)	3 (1,5)
*P*_4_	<0.001	<0.001	<0.001

*Note*: *P*_1_, *P*_2_, *P*_3_, *P*_4_: after 3, 6, 9, and 12 months of treatment vs. before treatment, respectively. Non‐normally distributed measurement data were depicted as the median (minimum, maximum), with intergroup comparisons conducted using the Kruskal–Wallis test.

### 3.2 Comparisons of pre‐ and post‐treatment inflammatory cytokine levels in AR patients

**FIGURE 1:**
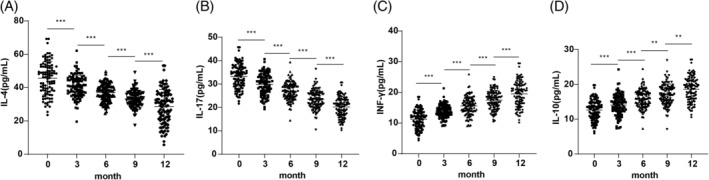
Comparisons of inflammatory cytokine levels in AR patients before and after treatment. Comparisons of serum IL‐4 (A), IL‐17 (B), INF‐γ (C), and IL‐10 (D) levels pre‐ and post‐treatment. Normally distributed measurement data were expressed as mean ± SD. Intergroup comparisons between different time points were analyzed by repeated measures ANOVA. **p <0.01, ***p <0.001.

Inflammatory cytokines, including IL‐4, IL‐17, INF‐γ, and IL‐10, were analyzed before and after SLIT treatment. IL‐4 and IL‐17 levels showed significant reductions at 3, 6, 9 and 12 months post‐treatment compared to baseline (Figure 1A,B, all *p* <0.01). In contrast, INF‐γ and IL‐10 levels increased significantly across the same time points (Figure 1C,D, all *p* <0.01).

### 3.3 Comparison of immune function levels in AR patients pre‐ and post‐treatment

**FIGURE 2:**
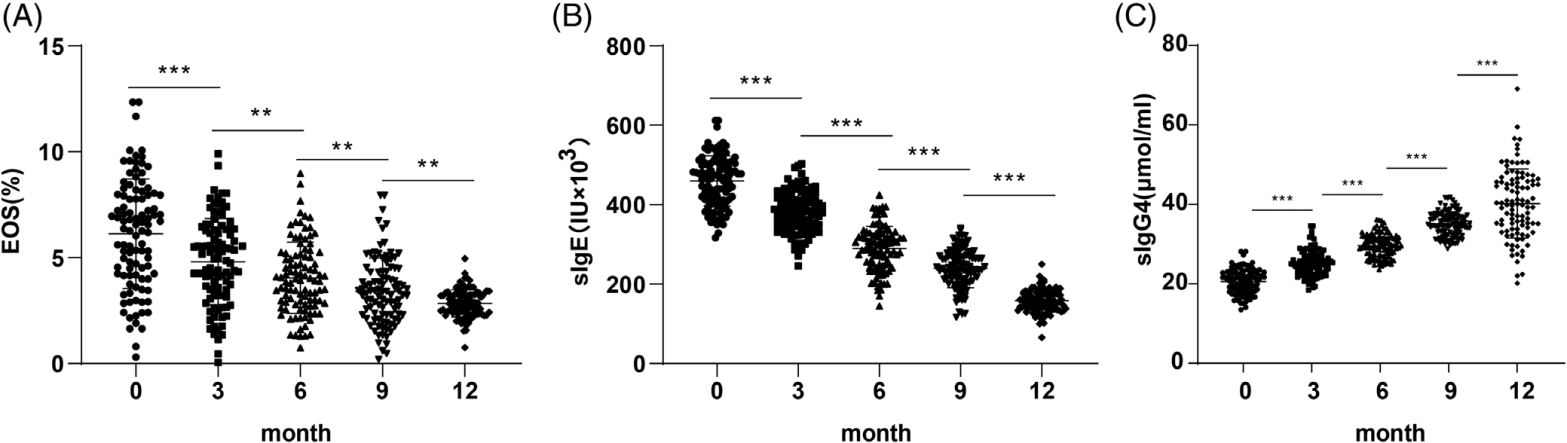
Comparison of immune function level in AR patients pre‐ and post‐treatment. Comparisons of ESO% (A), sIgE level (B), and sIgG4 level (C). Normally distributed measurement data were expressed as mean ± SD, and intergroup comparisons between different time points were analyzed using repeated‐measures ANOVA. **p <0.01.

We further evaluated immune function by comparing EOS%, sIgE, and sIgG4 levels before and after SLIT treatment. EOS% and sIgE levels significantly decreased following treatment (Figure 2A,B, all *p* <0.01), while sIgG4 levels increased substantially at each assessment point (Figure 2C, all *p* <0.01) suggesting enhanced immune tolerance associated with SLIT.

### 3.4 Correlation analysis on sIgG4 levels with TNSS, TMS, and VAS scores in AR patients pre‐ and post‐treatment

**FIGURE 3:**
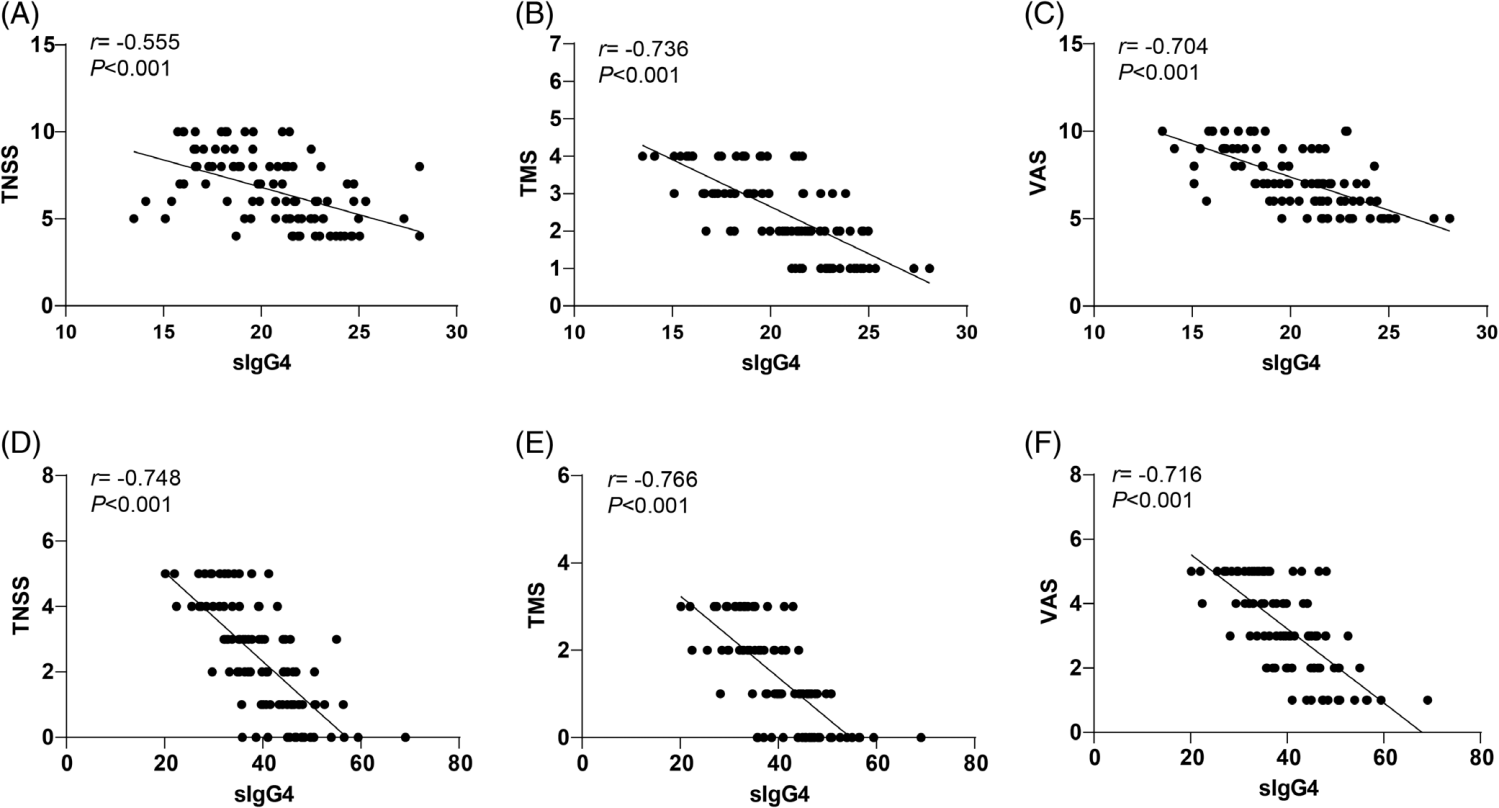
Correlation analysis of sIgG4 level with TNSS, TMS, and VAS scores. Spearman's correlation analysis was used to analyze the correlations of pre‐treatment sIgG4 level with TNS (A), TMS (B), and VAS scores (C), and the correlations of sIgG4 level with TNS (D), TMS (E), and VAS scores (F) after 12 months of treatment.

We examined the relationship between sIgG4 levels and TNSS, TMS, and VAS scores in AR patients before and after treatment. Spearman's correlation analysis indicated that, prior to treatment, sIgG4 levels were inversely correlated with TNSS (*r* = −0.555, *p* < 0.01), TMS (*r* = −0.736, *p* < 0.01), and VAS scores (*r* = −0.704, *p* < 0.01) (Figure 3A–C). At 12 months post‐treatment, sIgG4 levels showed a stronger inverse correlation with TNSS (*r* = −0.748, *p* < 0.01), TMS (*r* = −0.766, *p* < 0.01), and VAS scores (*r* = −0.716, *p* < 0.01) (Figure 3D–F), suggesting that higher sIgG4 levels were associated with improved clinical outcomes.

### 3.5 Correlation analysis of sIgG4 levels with inflammatory factors and immune function indicators

**FIGURE 4:**
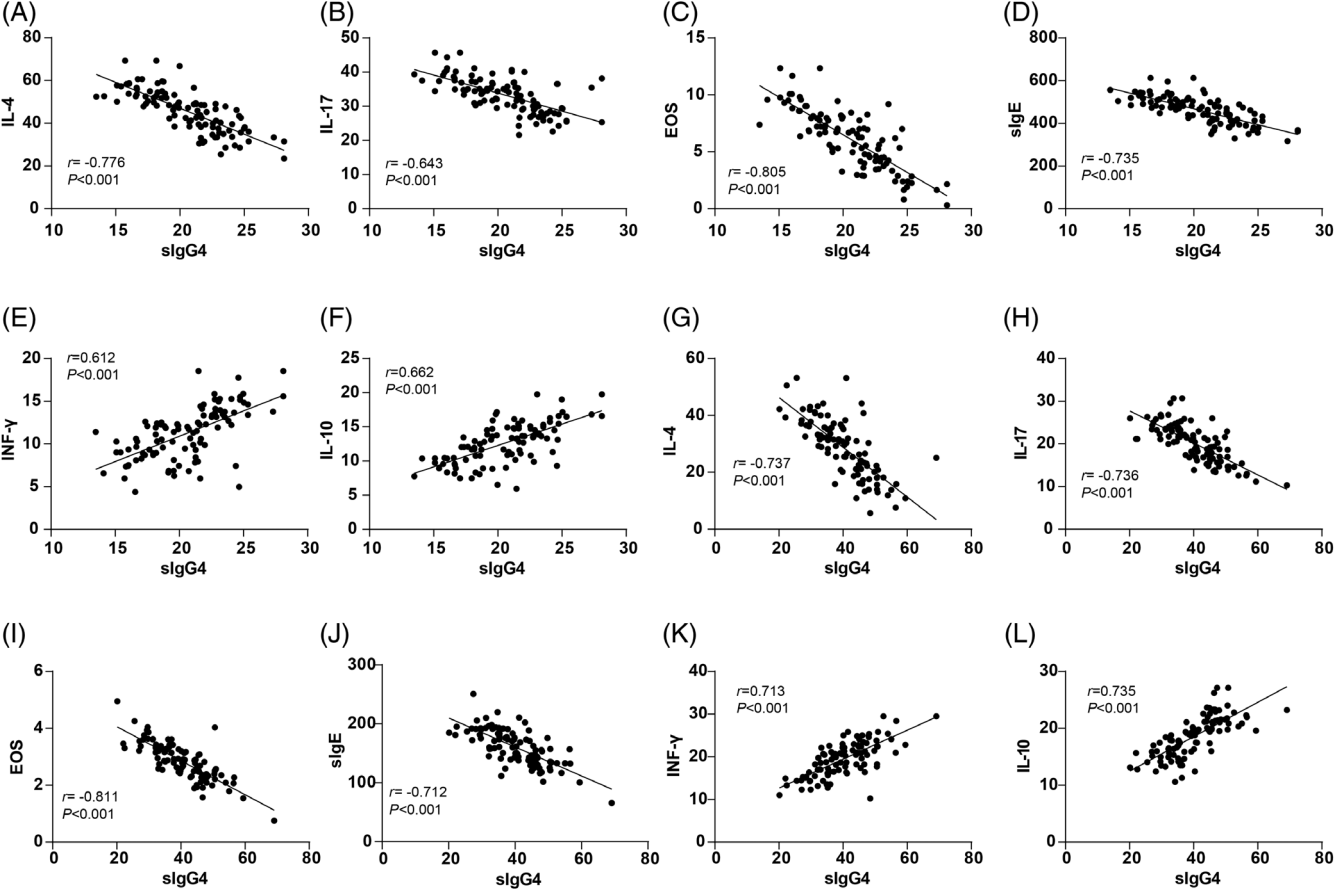
Correlation analysis of sIgG4 level with inflammatory factors and immune functions. Pearson's correlation coefficient was used to analyze the correlations of sIgG4 level with IL‐4, IL‐17, INF‐γ, IL‐10, ESO%, and sIgE before treatment (A–F) and after 12 months of treatment (G–L).

The correlations between sIgG4 levels and inflammatory factors (IL‐4, IL‐17, INF‐γ, IL‐10) as well as immune function indicators (EOS%, sIgE) were also assessed. Pearson's correlation analysis revealed that pre‐treatment sIgG4 levels were negatively correlated with IL‐4 (*r* = −0.776, *p* < 0.01), IL‐17 (*r* = −0.643, *p* < 0.01), EOS (*r* = −0.805, *p* < 0.01), and sIgE (*r* = −0.735, *p* < 0.01), while positively correlated with INF‐γ (*r* = 0.612, *p* < 0.01) and IL‐10 (*r* = 0.662, *p* < 0.01) (Figure 4A–F). After 12 months of treatment, sIgG4 levels maintained these correlations, showing significant negative correlations with IL‐4 (*r* = −0.737, *p* < 0.01), IL‐17 (*r* = −0.736, *p* < 0.01), EOS (*r* = −0.811, *p* < 0.01), and sIgE (*r* = −0.712, *p* < 0.01), and positive correlations with INF‐γ (*r* = 0.713, *p* < 0.01) and IL‐10 (*r* = 0.735, *p* < 0.01) (Figure 4G–L). These findings suggest that higher sIgG4 levels may be linked to a favorable immunological profile in AR patients undergoing SLIT.

### 3.6 Assessment of SLT efficacy in AR patients based on indicator levels after 12 months of treatment

**FIGURE 5:**
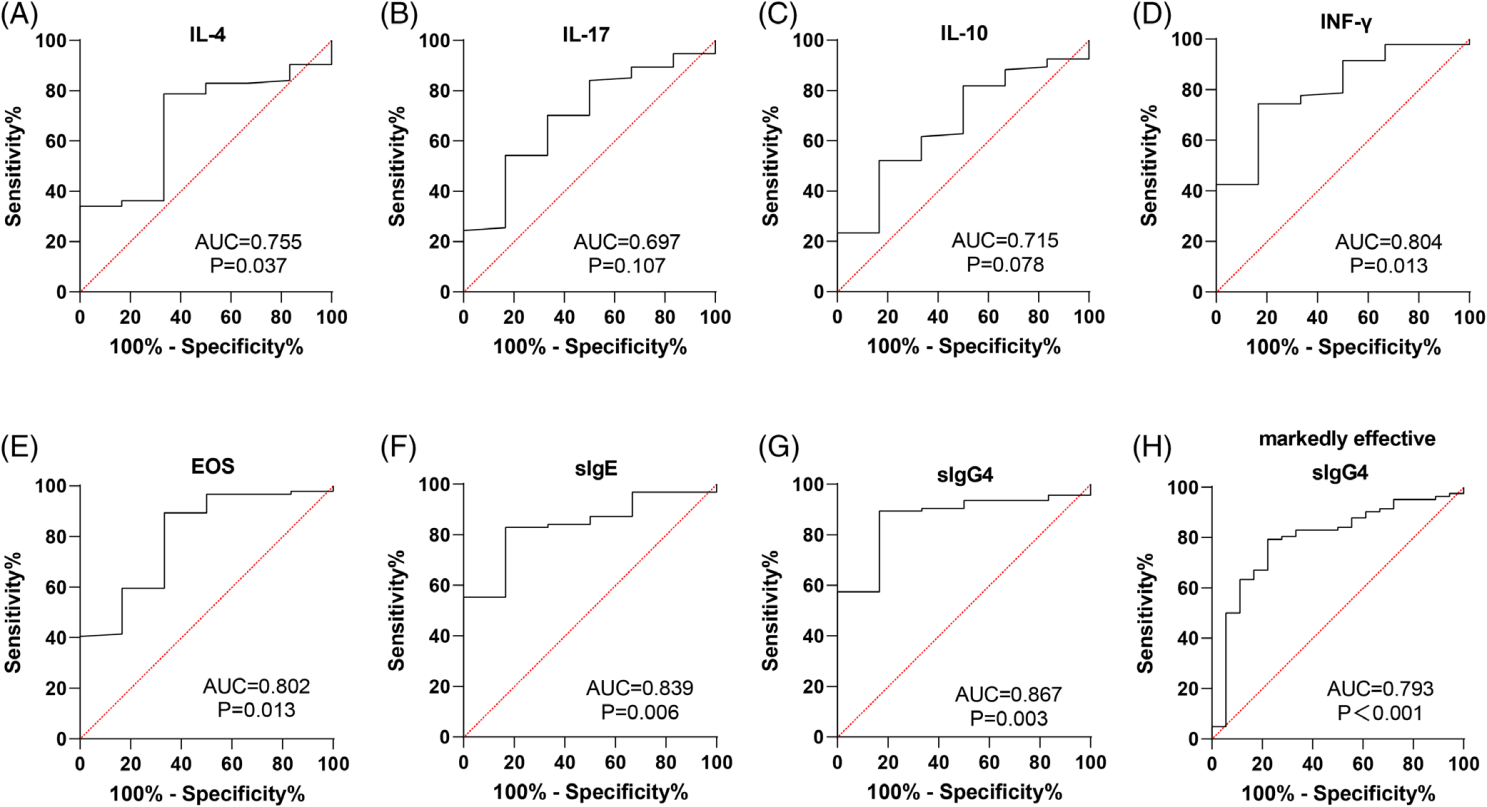
Analysis of the value of different indicators in assessing SLIT efficacy in AR patients after 12 months of treatment. (A–G) ROC curves to analyze the value of IL‐4 (A), IL‐17 (B), IL‐10 (C), INF‐γ (D), EOS (E), sIgE (F), and sIgG4 (G) levels for assessing effective efficacy in AR patients after 12 months of SLIT treatment, respectively; (H) ROC curves to analyze the value of sIgG4 levels in assessing markedly effective efficacy in AR patients.

SLIT efficacy was assessed by symptom scores, with an overall effective rate of 94.00% in AR patients (Table 2). ROC curve analysis evaluated the levels of IL‐4, IL‐17, IL‐10, INF‐γ, EOS, sIgE, and sIgG4 after 12 months to determine their effectiveness in assessing SLIT efficacy. As shown in Figure 5 and Table 3, INF‐γ, EOS, sIgE, and sIgG4 levels were significant indicators for assessing effective efficacy in AR patients (all *p* <0.05), with sIgG4 demonstrating the highest accuracy. At 12 months, sIgG4 had an area under the curve (AUC) of 0.867 (95% CI: 0.749–0.985, sensitivity of 89.36%, specificity of 83.33%, and a cut‐off value >31.69) for assessing SLIT efficacy. For cases classified as markedly effective, sIgG4 had an AUC of 0.793 (95% CI: 0.677–0.908, sensitivity of 79.27%, specificity of 77.78%, and a cut‐off value >35.26). The results suggest that sIgG4 levels at 12 months are reliable indicators for evaluating SLIT efficacy in AR patients.

**TABLE 2:** Efficacy evaluation of AR patients after SLIT treatment.

Markedly effective (cases)	Effective (cases)	Ineffective (cases)	Effective rate (%)
82	12	6	94

**TABLE 3:** ROC curve parameters of different indicators in assessing SLIT efficacy of AR patients after 12 months of treatment.

Parameter	Efficacy	AUC	95% CI	Sensitivity (%)	Specificity (%	Cut‐off value	*p* value
IL‐4	Effective	0.676	0.479 ~ 0.874	78.72	66.67	<36.06	0.149
IL‐17	Effective	0.697	0.495 ~ 0.899	54.26	83.33	<20.98	0.107
IL‐10	Effective	0.668	0.465 ~ 0.872	52.13	83.33	>19.05	0.168
IFN‐γ	Effective	0.804	0.642 ~ 0.966	74.47	83.33	>16.92	0.013
EOS	Effective	0.802	0.620 ~ 0.985	89.36	66.67	<3.505	0.013
sIgE	Effective	0.839	0.715 ~ 0.963	82.98	83.33	<190.1	0.006
sIgG4	Effective	0.867	0.749 ~ 0.985	89.36	83.33	>31.69	0.003
	Markedly effective	0.793	0.677 ~ 0.908	79.27	77.78	>35.26	<0.001

### 3.7 Predictive value of pre‐treatment sIgG4 levels for SLIT efficacy in AR patients

**FIGURE 6:**
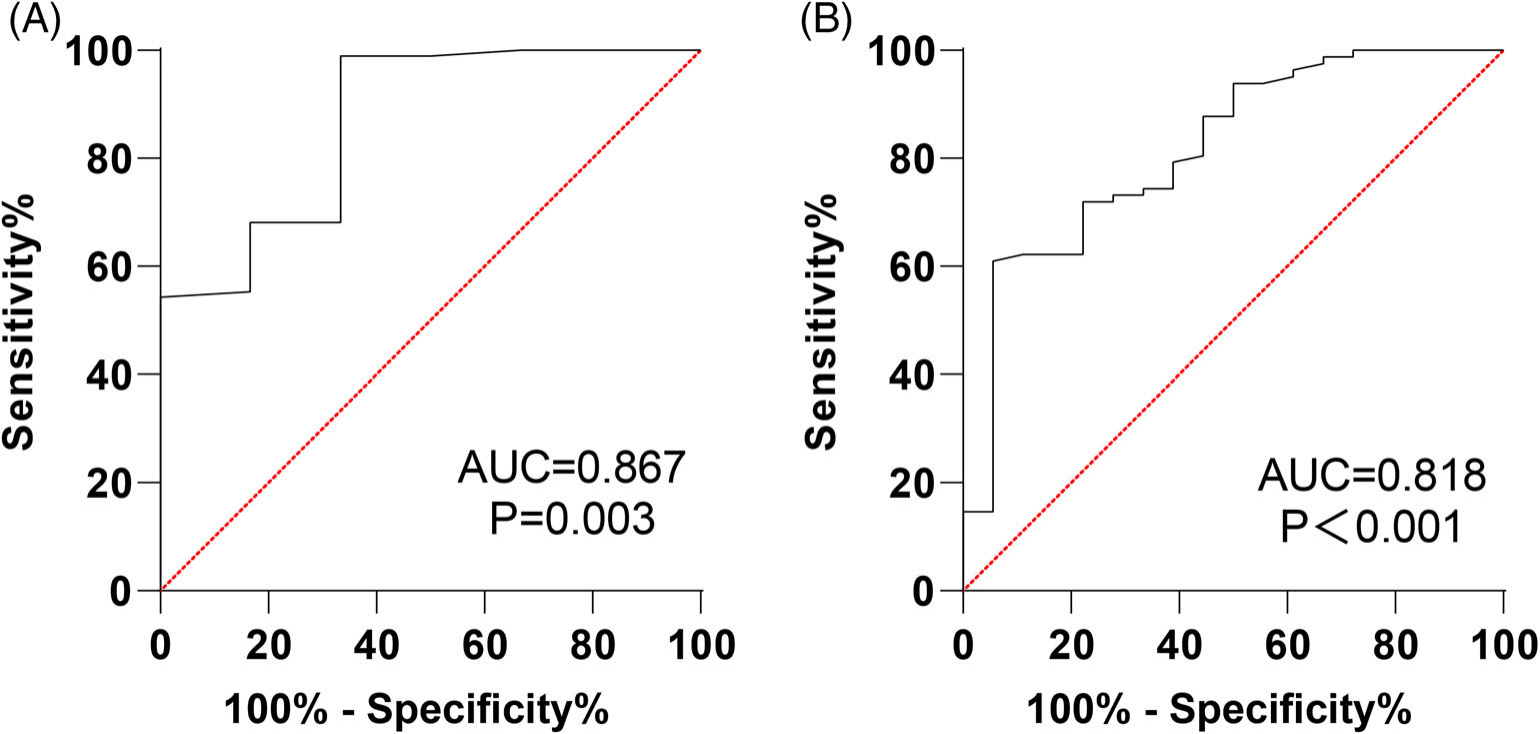
Analysis of the value of sIgG4 level in predicting SLIT efficacy in AR patients. ROC _effective_ curve (A) and ROC_markedly effective_ curve (B).

The predictive capability of pre‐treatment sIgG4 levels for SLIT efficacy was also examined through ROC curves. Pre‐treatment sIgG4 had an AUC of 0.869 (95% CI: 0.787–0.928, sensitivity of 66.67%, specificity of 98.94%, and a cut‐off value >15.42) for predicting SLIT as effective. For predicting marked effectiveness, the AUC was 0.818 (95% CI: 0.728–0.888, sensitivity of 94.44%, specificity of 60.98%, and a cut‐off value >20.74) (Figure 6 and Table 4). Overall, pre‐treatment sIgG4 levels provided a strong predictive value for the prognosis of SLIT efficacy in AR patients.

**TABLE 4:** ROC curve parameters of pre‐treatment sIgG4 level for predicting efficacy assessment of AR patients.

Efficacy	AUC	95%CI	Sensitivity	Specificity	Cut‐off value	*p* value
Effective	0.869	0.787 ~ 0.928	66.67	98.94	>15.42	0.003
Markedly effective	0.818	0.728 ~ 0.888	94.44	60.98	>20.74	<0.001

## 4 DISCUSSION

With over 30 years of clinical use, SLIT is well‐recognized for its disease‐modifying effects in AR, due to its established efficacy and favorable safety profile.^21^, ^22^, ^23^, ^24^, ^25^ Elevated sIgG4 levels are associated with symptom improvement and have emerged as immunological markers for assessing AR treatment.^12^, ^13^ This study examined the expression of sIgG4 in AR and analyzed its significance in evaluating SLIT efficacy. A primary innovation our research is the identification of correlations between sIgG4 levels, both pre‐ and post‐SLIT, with TNSS, TMS, and VAS scores, as well as inflammatory factors and immune function indicators in AR patients. Notably, we found that sIgG4 levels measured 12 months post‐treatment were valuable in assessing SLIT efficacy, while pre‐treatment sIgG4 levels showed predictive potential for treatment outcomes. This study therefore provides a novel reference for assessing SLIT's clinical efficacy in AR patients.

IgG4, a subclass of IgG antibody, exhibits unique properties, allowing it to bind bispecific antigens in a monovalent fashion and counteract inflammation effectively.^26^ Both sIgE and sIgG4 are produced by B lymphocytes in response to antigens and play significant roles in AR pathogenesis.^27^ In allergen‐specific immunotherapy, IgG4 competes with IgE for allergen binding sites, effectively inhibiting IgE‐mediated allergic responses.^12^ Studies have shown that SLIT reduces eosinophilic counts, total IgE levels, and medication needs.^28^ For instance, SLIT for grass pollen allergies has been associated with increased levels of allergen‐specific sIgG4, showing potential to modify AR progression.^29^ Our findings align with these observations, showing that SLIT treatment lowered EOS% and sIgE levels while increasing sIgG4 levels. We also found that sIgG4 levels correlated negatively with both EOS and sIgE levels pre‐ and post‐12 months of SLIT.

Subjective measures such as TNSS, TMS, and VAS scores are widely recommended for assessing SLIT efficacy.^7^, ^30^ A study by Shao et al. on children with house dust mite‐induced AR demonstrated significant improvements in TMS, TNSS, and VAS scores after 1 year of SLIT compared to controls.^31^ In addition, Lin et al. reported a 91.5% control rate (including well‐ and partially controlled patients) among AR patients after SLIT.^32^ Our study is consistent with these findings, showing marked reductions in TNSS, TMS, and VAS scores in AR patients following SLIT treatment. However, there is limited data on the relationship between sIgG4 levels and these symptom scores. Our study uniquely identified that both pre‐treatment and 12‐month post‐SLIT sIgG4 levels were negatively correlated with TNSS, TMS, and VAS scores, suggesting that sIgG4 could be a valuable indicator for SLIT efficacy in AR patients.

IL‐17 and IL‐4 are well‐established mediators in allergic inflammation, with elevated levels often observed in AR patients.^33^, ^34^ IL‐17, in particular, has been shown to be raised in AR patients, and SLIT studies report significant decreases in IL‐17 after 2–3 years of treatment.^35^ Similarly, IL‐4 reduction has been documented with allergen immunotherapy in seasonal AR patients.^36^ Meanwhile, IL‐10, a critical anti‐inflammatory cytokine, modulates inflammatory Th cells and maintains tissue homeostasis.^37^ Studies have shown significant increases in IL‐10 after 1 year of SLIT with house dust mites in AR patients.^38^ IFN‐γ, known for its anti‐inflammatory, antiviral, and immunomodulatory functions, is essential for infection control, and elevated IFN‐γ levels post‐SLIT have been associated with positive treatment responses in AR, often paired with a decreased IL‐4/IFN‐γ ratio.^39^

In line with previous findings, our study showed reductions in IL‐4 and IL‐17 and increases in INF‐γ and IL‐10 following 3, 6, 9, and 12 months of SLIT compared to pre‐treatment levels. Additionally, we found that sIgG4 levels were inversely related to IL‐4 and IL‐17, while positively correlated with INF‐γ and IL‐10, both before and after 12 months of SLIT. ROC curve analysis indicated that IL‐4, IL‐17, IL‐10, INF‐γ, EOS, sIgE, and sIgG4 levels all held value for assessing SLIT efficacy after 12 months, with sIgG4 demonstrating the highest performance. Specifically, the pre‐treatment sIgG4 level yielded an AUC of 0.869 (95% CI: 0.787–0.928, 66.67% sensitivity, 98.94% specificity, and cut‐off value >15.42) for predicting effective SLIT outcomes and an AUC of 0.818 (95% CI: 0.728–0.888, 94.44% sensitivity, 60.98% specificity, and cut‐off value >20.74) for predicting marked efficacy, underscoring its strong predictive value.

In sum, this study highlights that sIgG4 levels, both prior to and following 12 months of SLIT, correlated with clinical and immunological indicators, suggesting an association with AR disease severity. Increases in sIgG4 post‐SLIT may indicate disease remission, making it a useful measure of SLIT efficacy. Additionally, pre‐treatment sIgG4 levels demonstrated strong predictive potential regarding SLIT treatment success, supporting its role as a valuable marker in AR management.

sIgG4 shows promise as a biomarker for evaluating SLIT efficacy in AR patients. First, measuring blood sIgG4 is a noninvasive method that is generally well‐accepted by patients. Second, elevated sIgG4 levels are typically associated with effective SLIT treatment, displaying high sensitivity to therapeutic responses and accurately reflecting immune responses to specific allergens, lending sIgG4 high specificity. Additionally, tracking sIgG4 levels over time allows for monitoring SLIT progress, providing clinicians with valuable information to tailor treatment plans to individual patient needs.

However, sIgG4 also has notable limitations as a biomarker for SLIT efficacy. Baseline sIgG4 levels can vary significantly among individuals, and response to SLIT may also differ between patients, meaning changes in sIgG4 levels may not fully capture treatment efficacy for everyone. The lack of standardized detection methods and reference ranges complicates cross‐study comparisons, and symptom improvement may also be influenced by factors beyond sIgG4 changes. Thus, sIgG4 levels alone may not reflect clinical symptom improvement. In clinical practice, it is essential to consider sIgG4 in combination with other clinical indicators to gain a more comprehensive assessment of SLIT efficacy.

In summary, this study underscores that sIgG4 levels hold considerable value in assessing SLIT efficacy in AR patients, with level changes correlating with clinical scores (TNSS, TMS, and VAS) and immunological markers (IL‐4, IL‐10, IL‐17, INF‐γ, EOS%, and sIgE). However, the study had some limitations. Sample collection spanned a substantial time period, which could have impacted consistency in measurements, and the relatively small sample size restricted the robustness of our analysis. Future research should aim to expand the sample size and incorporate multicenter studies to deepen our understanding of sIgG4's role in evaluating SLIT efficacy in AR patients.

## CONFLICTS OF INTEREST STATEMENT

The authors declare no conflicts of interest.

## ETHICS STATEMENT

This study was approved by the Academic Ethics Committee of The Second Affiliated Hospital of Xiamen Medical College. Written informed consent was obtained from all participants. The study adhered to the ethical standards of the Declaration of Helsinki of the World Medical Association, as well as relevant norms and regulations, and followed the Enhancing the QUAlity and Transparency of Health Research (EQUATOR) network guidelines for enhancing the quality and transparency of health research.

## Data Availability

Data supporting the findings of this study are available from the corresponding author upon reasonable request.
